# Supplementary material for: Arylphthalide Delays Diabetic Retinopathy via Immunomodulating the Early Inflammatory Response in an Animal Model of Type 1 Diabetes Mellitus
Source: Int J Mol Sci. 2024 Aug 2;25(15):8440. doi: 10.3390/ijms25158440 (PMC11313200; doi:10.3390/ijms25158440)
Supplement: Supplementary file 1 [file ijms-25-08440-s001.zip › ijms-3113980-Supplementary.pdf]

Table S1: List of antibodies

Antibody	Company	Reference	Technical Approach	Concentration
α-Tubulin	Sigma-Aldrich (St. Louis, USA)	T5168	WB	1:10000
Phospho-p38α MAPK	Cell Signaling Technology (Danvers, USA)	9211S	WB	1:1000
p38α MAPK	Cell Signaling Technology (Danvers, USA)	9212S	WB	1:1000
Phospho-JNK	Cell Signaling Technology (Danvers, USA)	9255S	WB	1:1000
JNK	Cell Signaling Technology (Danvers, USA)	9252S	WB	1:1000
iNOS	Abcam (Cambridge, UK)	AB15323	WB	1:1000
HO-1	Abcam (Cambridge, UK)	AB189491	WB	1:1000
Caspase-1	Abcam (Cambridge, UK)	AB179515	WB	1:1000
IL1β	Mybiosurce (San Diego, USA)	MBS821750	WB	1:1000
NLRP3	AdipoGene Life Sciences (Füllinsdorf, Switzerland)	AG-20B-0014-C100	WB	1:1000
Rabbit-Peroxidase	Sigma-Aldrich (St. Louis, USA)	A0545	WB	1:5000
Mouse-Peroxidase	Sigma-Aldrich (St. Louis, USA)	A2554	WB	1:5000
Arginase-1	BD Biosciences (Madrid, Spain)	610708	IF/WB	1:400/1:1000

GFAP	DAKO (Glostrup, Denmark)	Z0334	IF	1:500
p65-NFκB	Cell Signaling Technology (Danvers, USA)	8242S	IF	1:500
IBA-1	Fujifilm-Wako (Madison, USA)	019-19741	IF	1:500
Rabbit Alexafluor-488	Thermo Fisher (Waltham, USA)	A11034	IF	1:1000
Mouse Alexafluor-568	ThermoFisher (Waltham, USA)	A11004	IF	1:800
DAPI	ThermoFisher (Waltham, USA)	D9542	IF	1:2000

Table S2. List of Taqman probes used for mouse and rat.

Gene	*Mus musculus*	*Rattus norvergicus*
Nos2	Mm00440502_m1	Rn00561646_m1
Il1b	Mm00434228_m1	Rn00580432_m1
Tnfa	Mm00443258_m1	Rn99999017_m1
Il6	Mm00446190_m1	Rn01410330_m1
Nlrp3	Mm00840904_m1	Rn04244620_m1
Arg1	Mm00475988_m1	Rn00691090_m1
ActineB	Mm00607939_s1	Rn00667869_m1
Hmox1	Mm*00516004_m1*	

Table S3: Average raw data Retinal thickness (μm)		
BB pre-treatment (7 weeks-old)	BB vehicle (9 weeks-old)	BB+M9 (9 weeks-old)
216,821	210,113	215,178
213,38	211,945	219,849
215,458	214,494	221,717
213,987	212,419	219,463
217,234	206,718	218,038
216,165	210,7299	213,721
215,999	196,0267	217,196
219,121	210,8943	218,107
216,557	204,0519	
213,987	210,8051	
221,9563	212,4261	
225,7026	212,3664	
227,9269	207,3965	
212,806	208,555	
237,1172	203,2374	
242,7545	205,3671	
229,5478		
220,5265		

Supplementary Materials

Reagents

Fetal bovine serum (FBS) and culture media were obtained from Invitrogen (Grand Island, NY, USA). Bovine serum albumin (BSA), crystal violet, glutaraldehyde, *N*-(1-naphthyl) ethylenediamine (NEDA), sulfanilamide, Triton X-100, sucrose, dimethylsulfoxide (DMSO), bacterial lipopolysaccharide (LPS), sodium dodecyl sulfate (SDS), penicillin/streptomycin and DL-Dithiothreitol (DTT) were purchased from Sigma-Aldrich (St. Louis, MO, USA). Protease inhibitors cOmplete-EDTA free from Roche (Madrid, Spain). Accutase was purchased from BioLegend (San Diego, CA, USA). Acrylamide and immunoblot PVDF membranes were purchased from Bio-Rad (Madrid, Spain). The BCA reagent and the cell culture inserts with a pore size 0.4 μm were purchased from Thermo Fisher (Whaltham, MA, USA), and Fluoromount-G was obtained from Southern Biotech (Alabama, USA). Thiobarbital 0,5 g was obtained from Braun Medical, S.A. (Rubí, Barcelona, Spain). L-glutamine was purchased from Gibco (Carlsbad, CA, USA).

Antibodies

Please see the table S1.

Cell culture

The mouse microglial cell line Bv.2 was purchased from ACCEGEN Biotechnology (ACCEGEN Biotechnology, Fairfield, USA). The mouse macrophages cell line Raw264.7 was provided by Dr. Valverde (IIBm "Alberto Sols" UAM-CSIC-Madrid, Spain). A total of 1.5 x 10^5 cells were seeded per well in a 6-wells plate (La Roca del Vallés, Barcelona, Spain). The cells were cultured at 37 °C in a humidified atmosphere with 5% CO_2 in RPMI supplemented with 10% (v/v) heat-inactivated FBS, 1% (v/v) penicillin/streptomycin and 2 mM L-glutamine. Both cell lines were grown to 80% confluence at which time they were washed twice with PBS and detached using accutase. Cells were used for experiments at passages 12-22.

Analysis of the Cellular Viability by Crystal Violet Staining

The cells were cultured in serum-free media and stimulated in the presence or absence of M9 (0.1, 0.5, 1, 10, 25 μM) for 24 hours to assess viability using crystal violet staining (PMID: 33815384). After cell treatments for 24 h, the media were discarded and the remaining viable adherent cells were fixed with 10% glutaraldehyde and stained with crystal violet (0.1% w/v in water) for 20 min. The plates were then rinsed with tap water and allowed to dry. Acetic acid (10%) was added to solubilize the crystal violet. The absorbance of each plate was read spectrophotometrically at 590 nm.

Analysis of Nitrites (NO_2^-)

To determine the nitrites production through the Griess test [42], the cells were stimulated with LPS (200 ng/mL) which mimics the diabetic proinflammatory environment (PMID: 27267343) in the presence or absence of M9 for 24 hours. Briefly, nitrites turn into pink color in contact with an acid solution containing 1% sulfanilamide and 0.1% *N*-(1-naphthyl) ethylenediamine (NEDA) and it was quantified by colorimetry at 540 nm in a microplate reader (PowerWave, Bioteck, Torino, Italy). In additional experiments, Bv.2 and Raw264.7 cells were cultured in a co-treatment regimen with M9 (10 μM) and LPS (200 ng/mL) for 24 hours.

Immunofluorescence

Bv.2 microglial cells were seeded on coverslips 24 h before LPS stimulation and/or M9 treatment in serum-free media. Cytosolic or nuclear P65 NFkB immunolocalization was determined as previously has been reported (PMID: 29191728). Briefly, the cells were washed in PBS, fixed with 4% (w/v) paraformaldehyde in Phosphate Buffer Saline (PBS) for 10 min at room temperature, washed in PBS, and permeated with 0.4% (v/v) Triton X-100 in PBS for 20 min. Blocking in PBS containing 3% (w/v) BSA and 0.1% (v/v) Triton X-100 for 2 h and, the cells were then left overnight in a humid chamber at 4°C with rabbit anti-P65 NFkB antibody (1:500) in blocking buffer (TBS containing 3% (w/v) BSA and 1% (v/v) Triton X-100). After that, the cells were incubated in the dark for 2 h with anti-rabbit conjugated AlexaFluor 488 antibody (ThermoFisher Scientific, Waltham, MA, USA). The nuclei were stained with 4,6-diamidino-2-phenylindole (DAPI, ThermoFisher Scientific, MA, USA) and mounted with Fluoromount G media from ThermoFisher Scientific (Waltham, MA, USA).

For retina immunofluorescence analysis, eye cryosections or whole retina explants were processing in similar way described previously (PMID: 27267343) for immunodetection of GFAP, IBA-1 and Arginase-1.

The eye cryosections were washed in TBS containing 0.1% (w/v) BSA and 0.1% (v/v) Triton X-100 (this buffer was used for all subsequent washes), and then permeabilized 30 min with TBS containing 3% (w/v) of Triton-X100 and then blocked for 2 h in TBS containing 3% (w/v) BSA and 1% (v/v) Triton X-100. The sections were then incubated overnight in a humid chamber at 4°C with rabbit anti-GFAP (1:500) or mouse anti-Arginase-1 (1:400) or rabbit anti-IBA-1 (1:500) antibodies in blocking solution. Sections were washed and incubated for 90 min at room temperature in a humid chamber in darkness, with anti-rabbit antibodies conjugated to AlexaFluor-488 or anti-mouse antibodies conjugated to AlexaFluor-568 (1:800, Molecular Probes, ThermoFisher Scientific, Waltham, MA, USA). After washing, sections were mounted with Fluoromount-G media containing DAPI.

For retinal explants immunofluorescence analysis, the whole retinas were fixed in 4% (w/v) paraformaldehyde for 24 h at 4°C and then, they were washed in PBS containing 0.1% (w/v) BSA and 0.1% (v/v) Triton X-100 (TBS) (this buffer was used for all subsequent washes), and blocked and permeated for 2 h in TBS containing 3% (w/v) BSA and 1% (v/v) Triton X-100. Subsequently, the retinal explants were incubated with a rabbit anti-GFAP antibody in blocking solution (1:500) overnight in a humid chamber at 4°C. Retinal sections and retinal explants were washed and incubated for 90 min with anti-rabbit immunoglobulin antibody conjugated to AlexaFluor 488 (1:1000). After washing, retinal explants were mounted with Fluoromount G media (Fluoromount G) containing DAPI and staining were observed and recorded with an inverted laser confocal microscope Axiovert (ZEISS, Jena, Germany).

Western Blot

Equal amount of proteins (20 µg) were resolved using denaturing SDS-PAGE, and transferred to PVDF membranes (Bio-Rad). Membranes were blocked using 5% skim milk or 3% BSA in PBS (10 mM Tris- HCl, 150 mM NaCl, pH 7.5), and incubated overnight at 4ºC with primary antibodies (1:1000 unless otherwise stated) in T-PBS (0.05% Tween-20-PBS). After, membranes were washed with T-PBS and incubated with the corresponding secondary peroxidase-conjugated antibody (1:2000) in blocking buffer for 2 h at room temperature. Blots were again washed with T-PBS and the immunoreactive bands were visualized using the Western-Bright Sirius reagent from Advansta Inc (San José, CA, USA) and a ChemiDoc™ Imaging System (Bio-Rad).

Quantitative Real-Time Polymerase Chain Reaction (qRT-PCR) Analysis

Total RNA was extracted with Trizol® reagent (Invitrogen, Madrid, Spain) and reverse transcribed using a iScript™ gDNA clear cDNA Synthesis kit (BioRad, Hercules, California, USA) for qPCR following the manufacturer's recommendations. qRT-PCRs were performed in a CFX96 Touch™ detection system from Bio-Rad laboratories. Mouse and rat Taqman probes for transcripts *Tnfa*, *Il6*, *Il1b*, *Il10*, *Nlrp3*, *Nos2*, *Arg1*, *Hmox1* and *Gapdh* (Table S 2) were purchased from Applied Biosystems (Waltham, MA, USA).

Intraperitoneal administration of M9

M9 was firstly dissolved in in DMSO to a stock concentration of 100 mM and then a working solution (600 µg/mL) were prepared in sterile conditions with normal saline solution and stored at 4 °C until further use. 10 female or male BB rats 7weeks-old age were randomly divided into M9 group and control group. Rats in M9 group were treated by intraperitoneal administration (i.p.) three days per week for two weeks (days 1–15) at the dosage of 600 µg/kg/day. The control group received the equal volume of vehicle in the same days. Blood glucose levels and body weight on days 0, 7 and 15 were determined and the eyeball or retinal tissue was processed for immunofluorescence, protein or RNA extraction.

On the day 16, the rats were euthanized by an overdose of anesthesia, and the eyes were enucleated. For immunofluorescence the eyes were fixed in 4% paraformaldehyde solution for 24 h at 4°C, cryoprotected with sucrose 25% (w/v) for another 24h, included in Tissue-Tek (Sakura Finetek, USA) and stored at -80°C until use. For protein or RNA extraction, the lens, anterior segment, vitreous humor, retinal pigment epithelium and sclera were removed and the retinas were immediately frozen at -80°C for protein or RNA extraction.

Spectral-domain optical coherence tomography (SD-OCT)

BB rats (7 week-old), BB rats vehicle (9 week old) and BB rats+M9 (9 weeks-old) were anaesthetized with ketamine (95 mg/kg) and xylazine (5 mg/kg) and maintained on a heated pad at 37°C. The eyes were instilled with a topical drop of 1% tropicamide (Colircusí Tropicamida, Alcon Cusí SA, Barcelona, Spain) for pupil dilation and 2% Methocel (Ciba Vision AG, Hetlingen, Switzerland) to prevent dryness and irritation. OCT images were obtained using a Micron IV rodent imaging system (Phoenix Research Labs, Pleasanton, CA, USA) as described previously [43]. A B-scan including the maximal retinal thickness (in the center of the retina) was segmented between the inner limiting membrane and the base of the retinal pigment epithelium using the Insight software package (Phoenix Research Labs). The thickness of both eyes (containing average raw data from 624 A-scans from each eye after removing the first and last 200 scans) was averaged per animal for statistical analysis. The overall average retinal layer thickness was presented as the mean ± standard error. The number of rats used in each measurement is described in Table S 3.

A

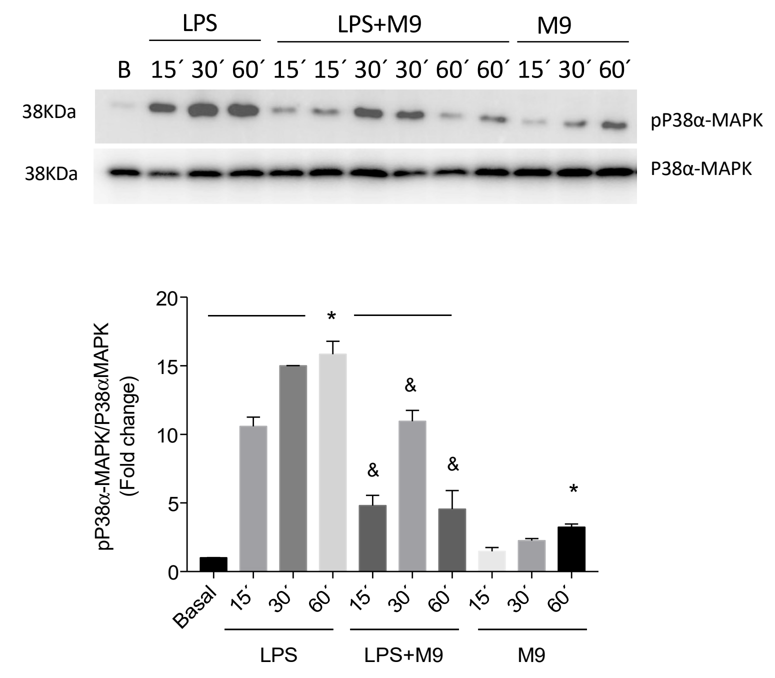
LPS
LPS+M9
M9
B 15´ 30´ 60´ 15´ 15´ 30´ 30´ 60´ 60´ 15´ 30´ 60´
38KDa
pP38α-MAPK
38KDa
P38α-MAPK
pP38α-MAPK/P38αMAPK (Fold change)
Basal
LPS
LPS+M9
M9

B

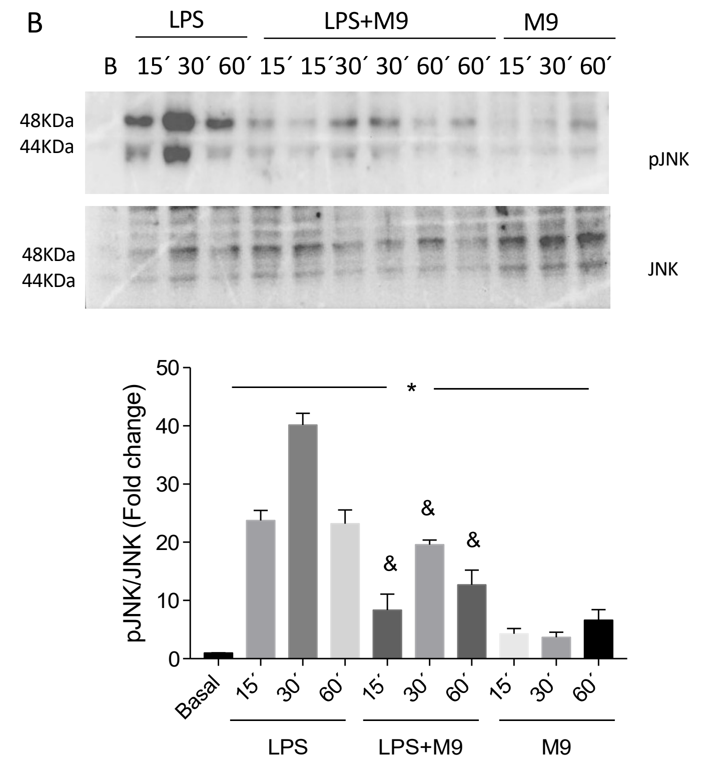
LPS
LPS+M9
M9
B 15´ 30´ 60´ 15´ 15´30´ 30´ 60´ 60´ 15´ 30´ 60´
48KDa
44KDa
pJNK
48KDa
44KDa
JNK
pJNK/JNK (Fold change)
Basal
LPS
LPS+M9
M9

Figure S 1

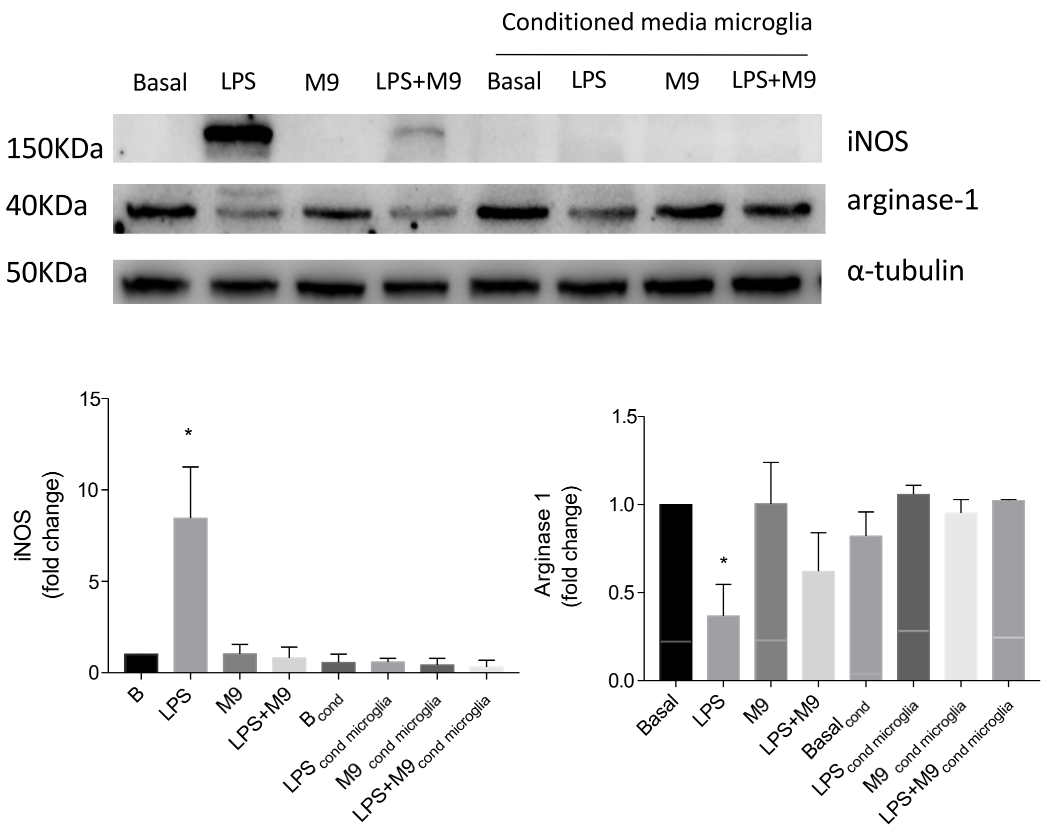
Conditioned media microglia
Basal
LPS
M9
LPS+M9
Basal
LPS
M9
LPS+M9
150KDa
iNOS
40KDa
arginase-1
50KDa
α-tubulin
iNOS (fold change)
15
10
5
0
*
B
LPS
M9
LPS+M9
B cond
LPS cond microglia
M9 cond microglia
LPS+M9 cond microglia
Arginase 1 (fold change)
1.5
1.0
0.5
0.0
*
Basal
LPS
M9
LPS+M9
Basal cond
LPS cond microglia
M9 cond microglia
LPS+M9 cond microglia

Figure S2

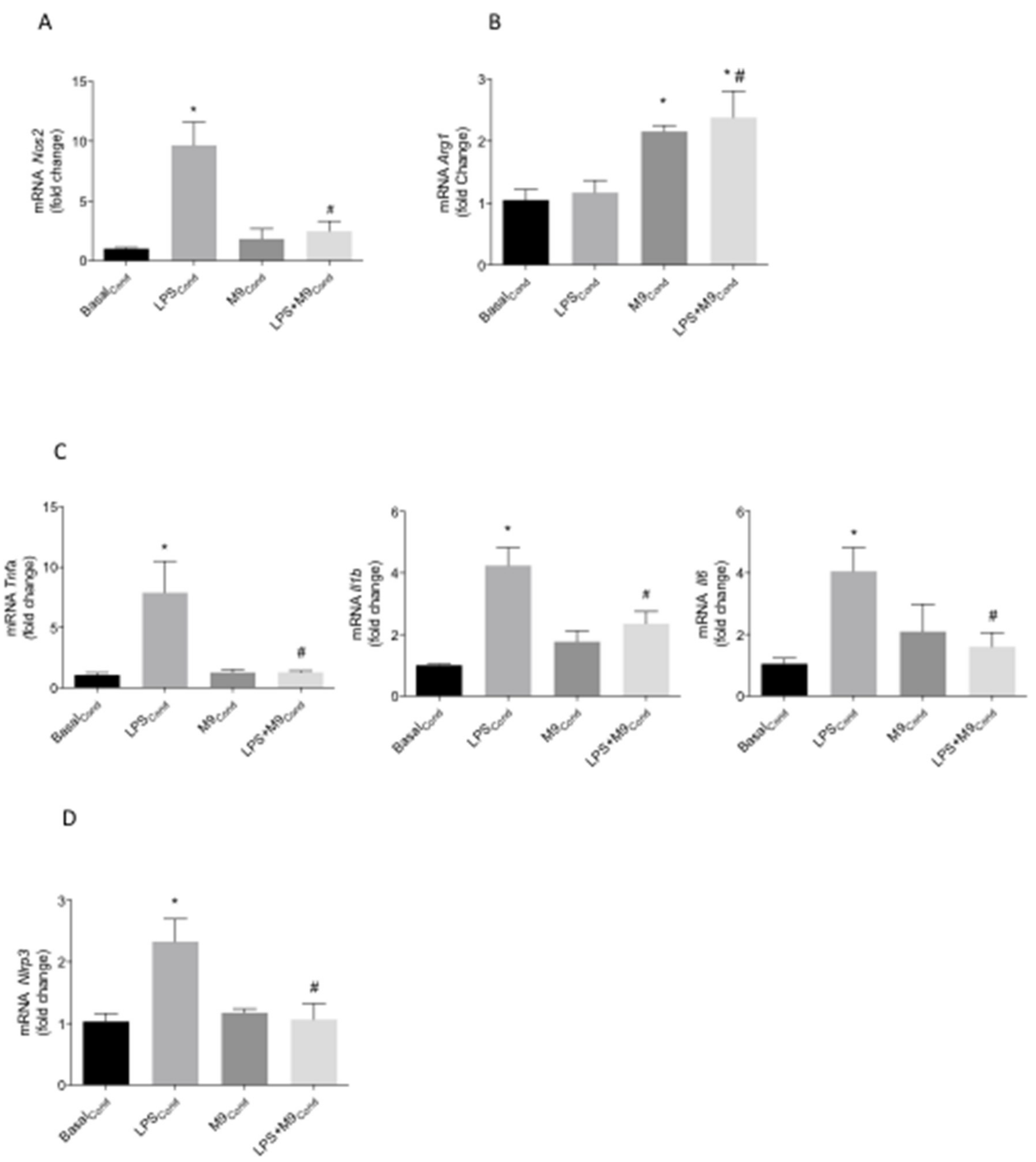
A
B
C
D
mRNA Nos2 (fold change)
mRNA Arg1 (fold Change)
mRNA Tnfa (fold change)
mRNA Il1b (fold change)
mRNA Il6 (fold change)
mRNA Nlrp3 (fold change)
Basal Cond
LPS Cond
M9 Cond
LPS+M9 Cond

Figure S3

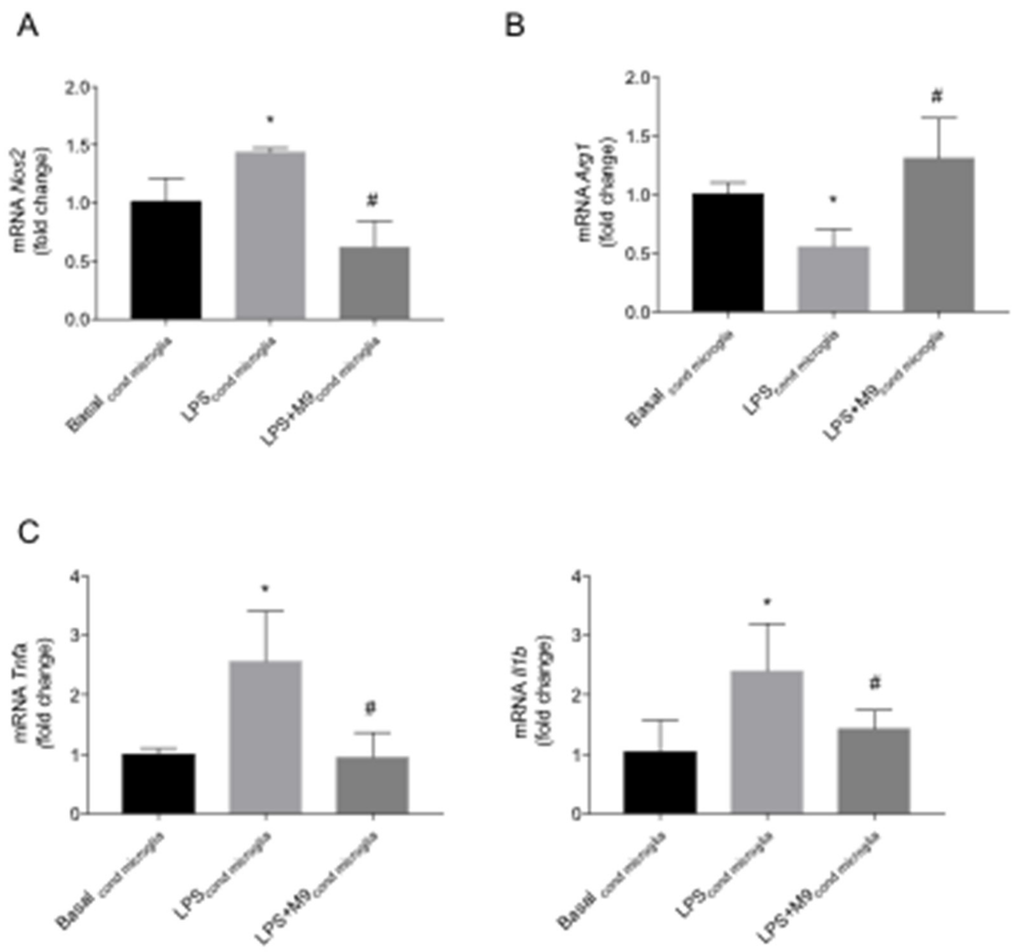
A
B
C
mRNA Nos2 (fold change)
mRNA Arg1 (fold change)
mRNA Tnfa (fold change)
mRNA Il1b (fold change)
Basal cond microglia
LPS cond microglia
LPS+M9 cond microglia
*
#

Figure S4

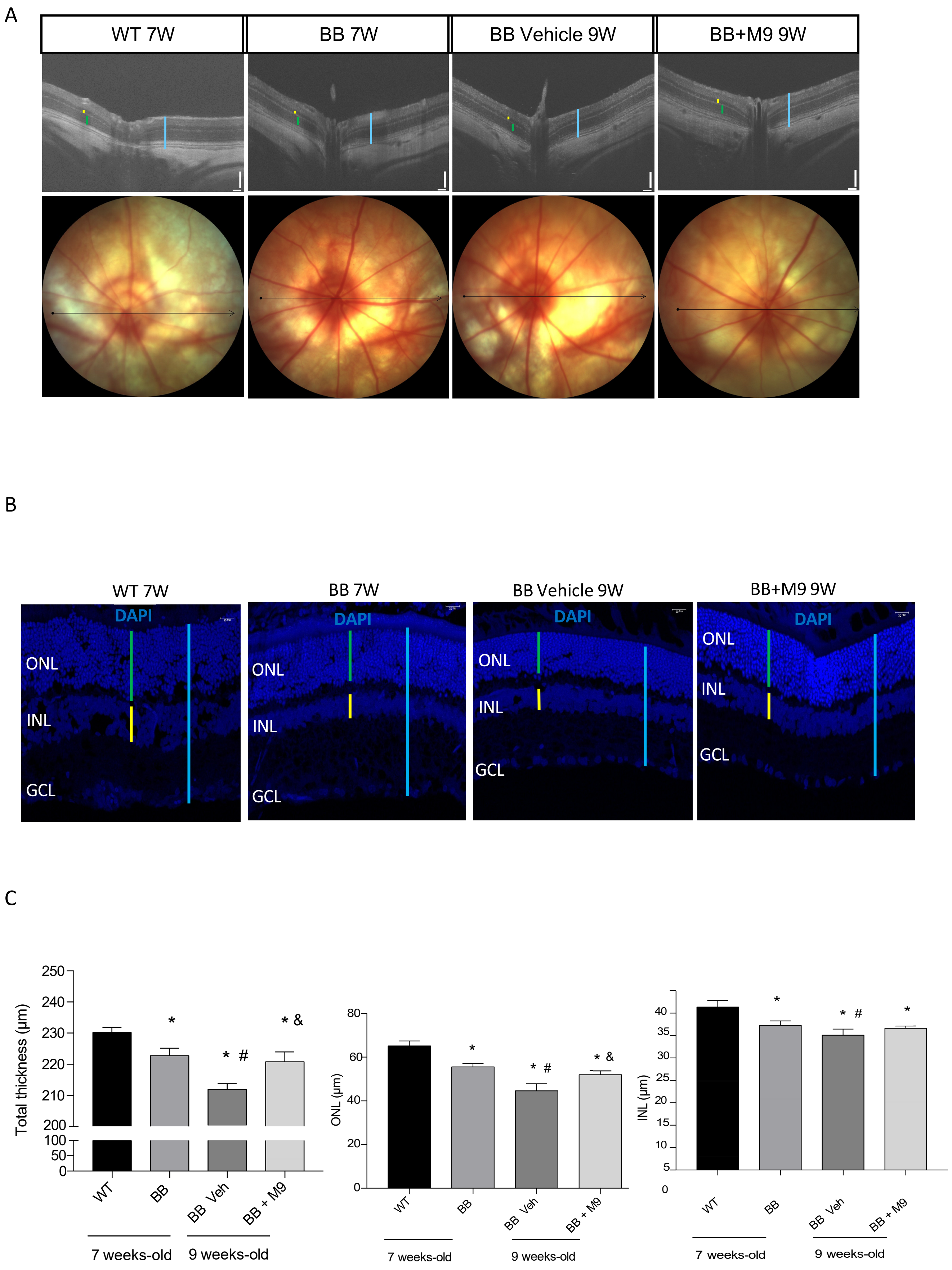

A
WT 7W
BB 7W
BB Vehicle 9W
BB+M9 9W
B
WT 7W
BB 7W
BB Vehicle 9W
BB+M9 9W
DAPI
ONL
INL
GCL
C
Total thickness (µm)
ONL (µm)
INL (µm)
WT
BB
BB Veh
BB + M9
7 weeks-old
9 weeks-old

Figure S5

Figure S1: M9 inhibited the activation of P38α-MAPK and JNK signalling in LPS- stimulated macrophage cells. Macrophage cells were treated for 24 h with LPS (200 ng/mL) or LPS plus M9 (10 μM) for the time-course indicated. **(A)** Protein extracts were analyzed by Western blot with antibodies against phosphorylated (p)-JNK, total JNK, **(B)** phosphorylated (p)-P38α MAPK, total P38α-MAPK. *α*-Tubulin was used as a loading control. Representative blots are shown (n=4 independent experiments). Blots were quantified with scanning densitometry, and the results are presented as mean ± S.E.M. The ratios between the indicated proteins and the fold changes relative to the basal values are shown. $^{*}p \le 0.05$ vs Basal treatment, $^{\&}p \le 0.05$ vs LPS treatment (one-way ANOVA followed by Bonferroni t-test).

Figure S2: Microglia conditioned medium does not induce any inflammatory/anti- inflammatory response in macrophage cells**.** Protein extracts were analyzed by Western blot with antibodies against iNOS and arginase-1. *α*- Tubulin was used as a loading control. Representative blots are shown (n=4 independent experiments). Blots were quantified with scanning densitometry, and the results are presented as mean ± S.E.M. The ratios between the indicated proteins and the fold changes relative to the basal values are shown. $^{*}p \le 0.05$ vs Basal treatment, $^{\&}p \le 0.05$ vs LPS treatment (one-way ANOVA followed by Bonferroni t-test).

Figure S 3: Macrophages conditioned medium induces the inflammatory response in retinas from WT rats but not promote the M2 response. Retinal explants from WT rats (7 weeks- old) were treated until 24 h with conditioned medium from Raw264.7 **(A)** *Nos2,* **(B)** *Arg1,* **(C)** *Tnfa, Il1b, Il6 and* **(D)** *Nlrp3* mRNA values were determined by qRT-PCR. The results are presented as mean ± S.E.M (n=5 retina per condition). Fold changes are calculated relative to the basal value. $^{*}p \le 0.05$ vs basal treatment, $^{\#}p \le 0.05$ vs LPS treatment (one-way ANOVA followed by Bonferroni t-test).

Figure S 4: Microglia conditioned medium induces the M1 and M2 response in retinas from WT rats. Retinal explants from WT rats (7 weeks-old) were treated until 24 h with conditioned medium from Bv.2 cells cultured previously with LPS (200 ng/mL) or LPS plus M9 (10 μM) for 24h. **(A)** *Nos2,* **(B)** *Arg1,* and **(C)** *Tnfa* and *Il1b* mRNA values were determined by qRT-PCR. The results are presented as mean ± S.E.M (n=5 retina per condition). Fold changes are calculated relative to the basal value. $^{*}p \le 0.05$ vs basal treatment, $^{\#}p \le 0.05$ vs LPS treatment (one-way ANOVA followed by Bonferroni t-test).

Figure S 5: (A)Representative SD-OCT images from the fundus of a WT, BB rats vehicle and BB rats M9 treatment. Arrows denote the B-scanned transects for thickness calculations, Blue line-1 (Total thickness), Green line-2 (ONL) and Yellow line-3 (INL). Scale bars = 100 μm. **(B)**DAPI histological eye sections from WT and BB rats treated with vehicle or M9. Arrows denote the B-scanned transects for thickness calculations. Blue-line = Total Thickness, Green line = ONL thickness and Yellow line = INL thickness. Scale bar= 20 μm. **(C)** Total, INL and ONL retinal thickness measured in DAPI dye sections. The results are presented as mean ± S.E.M. $^{*}p \le 0.05$ vs WT rat 7 weeks-old, $^{\#}p \le 0.05$ vs BB rat 7 weeks-old, $^{\&}p \le 0.05$ vs BB rat vehicle 9 weeks-old.
